# Investigating the Structural, Optical, and Thermal Properties of PVC/Cr_1.4_Ca_0.6_O_4_ Films for Potential Optoelectronic Application

**DOI:** 10.3390/polym17192646

**Published:** 2025-09-30

**Authors:** Alhulw H. Alshammari

**Affiliations:** Physics Department, College of Science, Jouf University, Sakaka P.O. Box 2014, Saudi Arabia; ahalshammari@ju.edu.sa

**Keywords:** Cr_1.4_Ca_0.6_O_4_, PVC, band gap energy, refractive index

## Abstract

This study demonstrates the successful preparation of pristine and modified PVC polymer films with (0.7, 1.0, 2.0, and 3.0 wt%) Cr_1.4_Ca_0.6_O_4_ by the solution casting method. These films were characterized using XRD, FTIR, XPS, SEM, TGA, and a UV–vis spectrophotometer. The XRD confirmed the amorphous nature of PVC films and a tetragonal zircon-type structure of Cr_1.4_Ca_0.6_O_4_ as a dopant in the PVC polymer. The XPS survey spectra of pristine Cr_1.4_Ca_0.6_O_4_ and its composites with PVC reveal essential insights into the materials’ surface composition and chemical states. The spectra clearly show peaks corresponding to O1s, Ca2p, and Cr2p, with the Cr2p signals being notably weaker than the other peaks. SEM images showed a uniform distribution of Cr_1.4_Ca_0.6_O_4_ within the PVC polymer films despite the presence of some minor agglomerations. The TGA analysis revealed that incorporating Cr_1.4_Ca_0.6_O_4_ enhanced the thermal stability of PVC films, particularly at a 0.7 wt% concentration of Cr_1.4_Ca_0.6_O_4_. Moreover, incorporation of Cr_1.4_Ca_0.6_O_4_ improved the optical parameters of PVC films, i.e., linear refractive index, nonlinear refractive index, and optical susceptibility. These findings proposed the modified PVC with Cr_1.4_Ca_0.6_O_4_ for optoelectronic applications.

## 1. Introduction

Polymers have outstanding properties such as electrical, structural, and physical properties; therefore, they have become desirable for widespread applications in diverse industries. Polymer nanocomposites have also attracted a broad range of applications due to their distinct properties [1,2]. Polyvinyl chloride is a well known polymer for its good properties, i.e., cost-effectiveness, easy fabrication [2,3,4,5], large surface area, high tensile strength, durability, as well as chemical stability [6]. Despite the aforementioned PVC polymer properties, it has some disadvantages such as poor thermal stability and brittleness, limiting its applications [2]. The thermal stability of PVC polymer can be reinforced when modified with suitable elements that have certain properties, i.e., metal oxides and carbon materials [7,8]. Meanwhile, other properties of modified PVC polymer, such as mechanical strength, biocompatibility, surface area, conductivity, etc., can be improved [9]. This improvement is attributed to the chemical and physical interaction between the PVC polymer and functionalized dopants that have good performance [7,8]. In addition, good dispersion of incorporated dopants within the PVC matrix has a positive impact on its performance [7]. Therefore, several studies have investigated the properties of modified PVC polymers with various elements [7]. The PVC/Cd_0.5_Zn_0.5_O films have been synthesized and their structural, optical and thermal properties have been investigated [10]. The inclusion of Cd_0.5_Zn_0.5_O enhanced the thermal stability of PVC film, while simultaneously decreasing the transparency of PVC film. Another study investigated the impact of PbO on the dielectric and optical properties of PVC [11]. Increasing PbO nanoparticle concentration increased the refractive index of the PVC films, while decreasing their direct band gaps and transparency. The effect of ZnO nanoparticles’ presence on the optical properties of PVC films was studied by Al-Taa’y et al. [12]. An increase in the content of ZnO nanoparticles has led to higher refractive index, optical conductivity, and dielectric constant values in PVC films. Another study exhibited the impact of ZnO on the properties of PVC films, i.e., thermal, thermomechanical, and morphological properties [7]. The thermal behavior, structural, and optical properties of Pb_3_O_4_/PVC nanocomposite films were studied [13]. The incorporation of Pb_3_O_4_ nanoparticles led to a reduction in the direct energy gap of PVC films. However, the Fermi energy and Urbach energy of PVC films were improved due to the additions of (0, 1, 2, 3, and 4 wt%) Pb_3_O_4_. The positive impact of graphene nanoplatelets (GNP) on the mechanical and thermal properties of PVC polymer [14]. Finally, a previous study highlighted the effect of Cr_2_O_3_ on the optical and dielectric properties of PVC film [15]. As the content of Cr_2_O_3_ increased, the direct energy gap, single oscillator energy, and transparency of PVC films decreased. Cr_1.4_Ca_0.6_O_4_ was selected as a dopant for PVC films due to its unique mixed metal oxide composition, combining chromium and calcium ions, which imparts both optical tunability and structural stability. Chromium-based oxides, such as Cr_2_O_3_, have previously demonstrated effectiveness in enhancing the optical and dielectric properties of polymer matrices by reducing the optical band gap and increasing refractive index and dielectric constant [15]. However, Cr_2_O_3_ alone can suffer from dispersion and aggregation issues at higher concentrations, which may limit its performance. The inclusion of calcium in the Cr_1.4_Ca_0.6_O_4_ structure provides improved dispersion and a tetragonal zircon-type phase, which facilitates better integration with the PVC matrix and enhances thermal and optical properties [16]. Although a direct experimental comparison was beyond the scope of this study, these prior results help contextualize the advantages of Cr_1.4_Ca_0.6_O_4_ as a multifunctional dopant. Future work will aim to benchmark Cr_1.4_Ca_0.6_O_4_-modified PVC films against other commonly used dopants under standardized conditions to further validate their potential in optoelectronic applications.

This study aims to fabricate PVC films doped with 0.7, 1.0, 2.0, and 3.0 wt% Cr_1.4_Ca_0.6_O_4_ using the solution casting technique. The incorporation of Cr_1.4_Ca_0.6_O_4_ led to a moderate enhancement in the thermal stability of the PVC films, particularly at lower doping levels, with 0.7 wt% showing the most notable improvement. In addition, a significant enhancement in optical properties was observed, including a reduced optical band gap and an increased refractive index, which supports the potential of these materials for optoelectronic applications.

## 2. Materials and Sample Preparations

The Polyvinyl chloride (PVC, average M_w_~233,000) was purchased from MERK, Germany, while tetrahydrofuran (THF) was purchased from CARLO ERBA. The chromium (III) nitrate (nonahydrate) 98%, Calcium nitrate tetrahydrate 98%, and gelatin powder were obtained from LOBA CHEMI, India.

The PVC/Cr_1.4_Ca_0.6_O_4_ was prepared by solution casting method. The Cr_1.4_Ca_0.6_O_4_ was prepared by dissolving 2 g of Cr(NO_3_)_3_⋅9H_2_O in 25 mL of distilled water and 0.708 g of Ca(NO_3_)_2_⋅4H_2_O in 25 mL of distilled water separately. These solutions were then mixed while stirring. In another beaker, 1.354 g of gelatin powder was also dissolved in distilled water using magnetic stirring for 60 min at 80 °C. Then, the metal nitrate solution was slowly added to the gelatin solution and stirred for 60 min at 7000 rpm. The solution was transferred to an oven adjusted to 240 °C for 120 min to evaporate the water. The final product was ground into fine powder. The fine powder was calcinated at 500 °C for 4 h inside a muffle furnace. The PVC/Cr_1.4_Ca_0.6_O_4_ films were prepared by dissolving 1 g of PVC polymer in 30 mL of THF under stirring for 60 min at room temperature. Different amounts of Cr_1.4_Ca_0.6_O_4_ (0.007, 0.010, 0.020, and 0.030 g) were added separately to the PVC solutions and stirred for 60 min. Finally, these solutions were poured into Petri dishes and left to dry at room temperature for 72 h, then peeled off to conduct some necessary characterization. The XRD patterns were obtained for pristine and modified PVC with Cr_1.4_Ca_0.6_O_4_ using a Shimadzu diffractometer (model 7000, Kyoto, Japan) with CuKα radiation (λ = 1.54056 Å) and a scan range of 2θ from 10° to 80°. FTIR spectra with a wavenumber range of 399 to 4000 cm^−1^ were obtained by ATR spectra using A Shimadzu spectrometer (FTIR–Tracer 100, Japan). X-ray photoelectron spectroscopy (XPS) was used for the prepared films using a K-alpha XPS spectrometer (Thermo Fisher Scientific, Waltham, MA, USA). The morphology of the prepared film was examined by A Thermo Fisher Quattro SEM (Thermo Fisher Scientific, Waltham, MA, USA). The cross-section of pristine and modified PVC films with Cr_1.4_Ca_0.6_O_4_ is approximately 160 µm. The mass loss as function of temperature in the range of 30–600 °C was tested using a Shimadzu TGA-51 Thermogravimetric Analyzer (Japan). Finally, light absorption in the wavelength range of 190–800 nm for the prepared polymer films was also measured using A Cary 60 UV–vis spectrophotometer, Agilent (Santa Clara, CA, USA).

## 3. Results and Discussion

**Figure 1:**
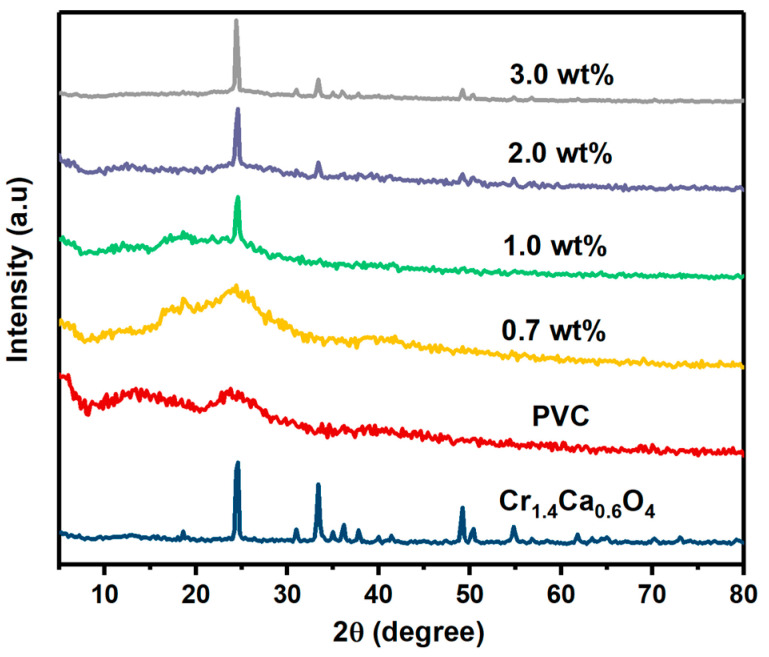
XRD Patterns of Pristine PVC and PVC Films Doped with 0.7, 1.0, 2.0, and 3.0 wt% Cr_1.4_Ca_0.6_O_4_.

X-ray diffraction (XRD) is a fundamental technique for analyzing the structural properties of materials. The XRD patterns of pristine PVC and PVC films doped with varying concentrations of Cr_1.4_Ca_0.6_O_4_ (0.7, 1.0, 2.0, and 3.0 wt%) are displayed in Figure 1 and Table 1. The pristine PVC film exhibits a broad peak centered around 2θ ≈ 24.6°, which is characteristic of its amorphous nature, as reported in previous studies [17,18,19]. In contrast, Cr_1.4_Ca_0.6_O_4_ displays sharp diffraction peaks at 2θ ≈ 18.59°, 24.61°, 30.98°, 33.40°, 34.99°, 36.24°, 49.22°, 50.40°, and 54.82°, corresponding to the (101), (200), (211), (112), (220), (202), (312), (400), and (004) crystal planes, respectively. These peaks are consistent with a tetragonal zircon-type crystal structure (JCPDS card No. 87-1647) [16]. In the doped PVC films, the diffraction peaks associated with Cr_1.4_Ca_0.6_O_4_ are not clearly visible at the 0.7 wt% loading, likely due to low dopant content or effective dispersion at the nanoscale. However, at higher loadings (1.0, 2.0, and 3.0 wt%), distinct Cr_1.4_Ca_0.6_O_4_ peaks emerge, confirming the successful incorporation of the oxide phase and indicating increased crystallinity with higher dopant concentration. The average crystal size of Cr_1.4_Ca_0.6_O_4_ was calculated by using Debye–Scherrer equation as follows:

(1) $$D=\frac{\;0.9\lambda }{\beta \;cos\;\theta }$$

where D and λ represent the crystallite size and X-ray wavelength, respectively, while β represents the full width at half maximum. As summarized in Table 1, the average crystallite size of pure Cr_1.4_Ca_0.6_O_4_ is approximately 30.52 nm, while that of the doped films ranges from 16.70 to 46.27 nm, depending on concentration. The FWHM and d-spacing values remain nearly constant across samples, but variations in crystallite size suggest a correlation between dopant loading and nucleation behavior during film formation. These results confirm the crystalline contribution of Cr_1.4_Ca_0.6_O_4_ and its structural interaction within the PVC matrix.

**Figure 2:**
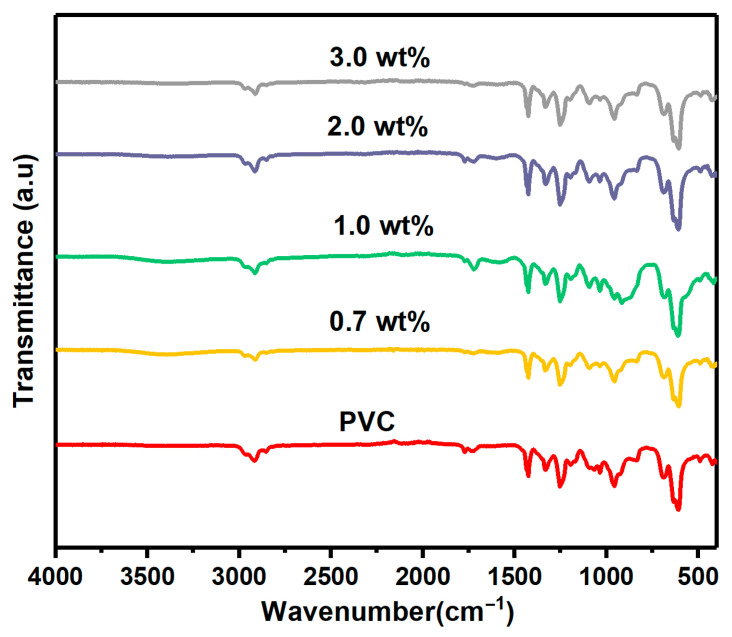
FTIR Spectra of Pristine PVC and PVC Films Doped with 0.7, 1.0, 2.0, and 3.0 wt% Cr_1.4_Ca_0.6_O_4_.

Figure 2 shows the FTIR spectra in the wavenumber range 400–4000 cm^−1^ for the pristine and modified PVC with 0.3, 0.7, 1.0, and 3.0 wt% Cr_1.4_Ca_0.6_O_3_. The located band at 1400 cm^−1^ is ascribed to C-H aliphatic bending bond, while peak at 1250 cm^−1^ is ascribed to C-H bending bond [20]. The appearing absorption bands between 1000 and 1100 cm^−1^ are due to the stretching bond of the C-C in the PVC backbone chain [20]. The stretching of C-Cl caused the absorption bands at 616 and 692 cm^−1^. Finally, stretching vibrations of C–C, CH_2_–Cl, C–H and CH_2_ caused the absorption bands at 1097, 1252, 2912 and 2971 cm^−1^, respectively [21]. Upon the inclusion of Cr_1.4_Ca_0.6_O_3_ into the PVC film, an absorption band that appeared at nearly 1067 cm^−1^ shifted to 1094 cm^−1^. This shift is attributed to the presence of a hydroxyl group linked to Cr^3+^ ions [16].

**Figure 3:**
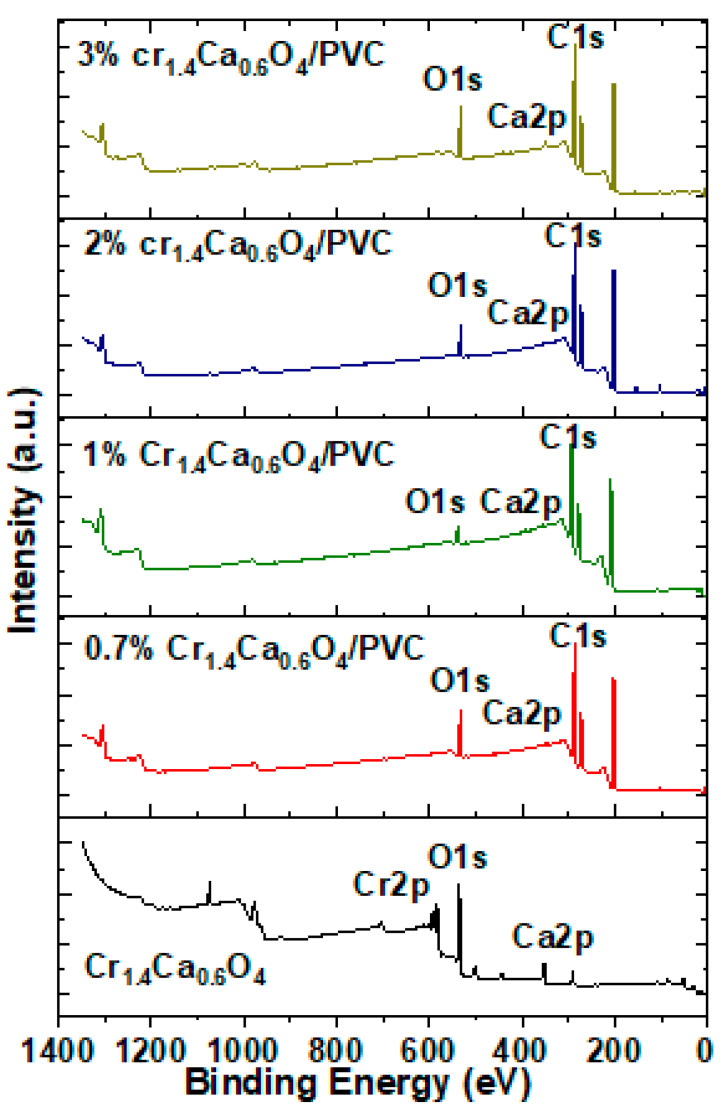
XPS Survey Spectra of Pristine Cr_1.4_Ca_0.6_O_4_ and PVC Films Doped with 0.7, 1.0, 2.0, and 3.0 wt% Cr_1.4_Ca_0.6_O_4_.

The XPS survey spectra of pristine Cr_1.4_Ca_0.6_O_4_ and its composites with PVC reveal essential insights into the materials’ surface composition and chemical states. The spectra clearly show peaks corresponding to O1s, Ca2p, and Cr2p, with the Cr2p signals being notably weaker than the other peaks (Figure 3). For pristine Cr_1.4_Ca_0.6_O_4_, the weak Cr2p intensity can be attributed to the surface sensitivity of XPS and the partial reduction or oxidation of chromium species on the material’s surface. This phenomenon is consistent with the behavior of chromium-based oxides, in which the oxidation state and distribution of chromium atoms may result in reduced signal intensity. In the Cr_1.4_Ca_0.6_O_4_/PVC composites, adding PVC introduces further attenuation of the Cr2p signal. As the concentration of Cr_1.4_Ca_0.6_O_4_ increases from 0.7% to 3%, the characteristic peaks of O1s, Ca2p, and C1s from the polymer matrix are observed; however, the Cr2p signal remains weak. This attenuation can be explained by the dilution effect caused by the non-conductive PVC matrix, which reduces the effective concentration of chromium at the surface region probed by XPS. Additionally, the polymer coating on Cr_1.4_Ca_0.6_O_4_ particles may limit the escape depth of photoelectrons from chromium atoms, further diminishing the observed Cr2p signal. The surface segregation of the polymer and preferential interaction of oxygen atoms with the PVC matrix could also suppress the Cr2p signal. These observations align with findings from the literature, where XPS signal attenuation in composite systems has been extensively documented. For example, Cimino et al. (1999) [22] demonstrated that surface coverage or segregation in supported oxide catalysts can significantly reduce photoelectron intensities. Similarly, Musiani (2000) [23] highlighted that polymer matrices in composite materials often obscure underlying metallic or oxide species, particularly when the polymer dominates the surface composition. Additional reviews on polymer composite systems further emphasize the role of matrix effects in reducing signal intensities, particularly for metallic oxides in non-conductive environments. These findings are consistent with established studies on the surface and bulk interaction of oxide/polymer composite systems.

**Figure 4:**
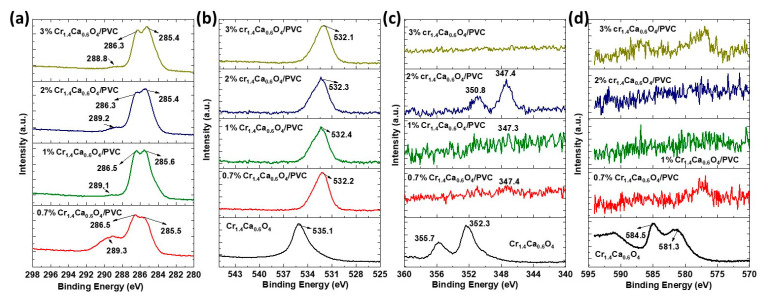
High-Resolution XPS Spectra of (**a**) C 1 s, (**b**) O 1 s, (**c**) Ca 2 p, and (**d**) Cr 2 p for Pristine Cr_1.4_Ca_0.6_O_4_ and PVC Films Doped with 0.7, 1.0, 2.0, and 3.0 wt% Cr_1.4_Ca_0.6_O_4_.

The high-resolution XPS spectra presented in Figure 4 provide detailed insights into the elemental composition and chemical states of pristine Cr_1.4_Ca_0.6_O_4_ and Cr_1.4_Ca_0.6_O_4_/PVC composites with varying concentrations of Cr_1.4_Ca_0.6_O_4_ (0.7%, 1.0%, 2.0%, and 3.0 wt%). The C1s spectra for all the composites exhibit peaks at binding energies of approximately 285.4 eV and 288.8 eV, which are attributed to the C–C bonds and carbonyl groups, respectively. The consistent appearance of these peaks indicates the dominant presence of PVC in the composites. With increasing Cr_1.4_Ca_0.6_O_4_ concentration, no significant shift or broadening of these peaks is observed, suggesting that the incorporation of Cr_1.4_Ca_0.6_O_4_ does not significantly alter the chemical environment of the carbon atoms in the PVC matrix. This result aligns with findings in studies of metal oxide/polymer composites, where the metal oxide phase predominantly interacts with other polymer matrix components rather than directly affecting carbon chemistry [24]. The O1s spectra show peaks around 532.2 eV for the Cr_1.4_Ca_0.6_O_4_/PVC composites, corresponding to oxygen atoms in the PVC matrix or oxygen associated with Cr_1.4_Ca_0.6_O_4_. For the pristine Cr_1.4_Ca_0.6_O_4,_ a higher binding energy peak is observed at approximately 535.1 eV, which may be attributed to oxygen in more strongly oxidized environments. This shift suggests that the PVC matrix influences the oxygen environment when the polymer incorporates Cr_1.4_Ca_0.6_O_4_, possibly through surface interactions or partial coverage. Such shifts in binding energy have been documented in studies of polymer/metal oxide composites, where the polymer matrix can alter the electron density around oxygen atoms [25]. The Ca2p spectra for the composites display distinct peaks at approximately 347.4 eV, consistent with calcium in the Cr_1.4_Ca_0.6_O_4_ phase. For the pristine material, additional features at higher binding energies, such as 350.8 eV, may indicate multiple oxidation states or calcium in different environments. The absence of these extra features in the composites suggests that the interaction with the PVC matrix homogenizes the calcium environment. This phenomenon is commonly observed in oxide/polymer systems where the matrix modifies the surface chemistry of embedded particles [22]. The Cr2p spectra exhibit weak signals at binding energies of approximately 581.3 eV and 584.5 eV for pristine Cr_1.4_Ca_0.6_O_4_, consistent with Cr^3+^ in the oxide. In the composites, the Cr2p signals are almost indistinguishable due to attenuation by the PVC matrix. This attenuation effect is well-documented in XPS studies of polymer composites, where non-conductive matrices reduce the effective photoelectron intensity from buried oxide phases [23]. The high-resolution XPS spectra clearly show the interactions between Cr_1.4_Ca_0.6_O_4_ and the PVC matrix. The weak Cr2p signals, shifts in O1s peaks, and changes in Ca2p features suggest that the PVC matrix significantly influences the surface chemistry of Cr_1.4_Ca_0.6_O_4_. These results are consistent with prior studies on oxide/polymer composites, which demonstrate the role of polymer matrices in altering the surface environment and attenuating XPS signals of embedded oxides. This understanding is critical for designing advanced composite materials with tailored properties for specific applications.

**Figure 5:**
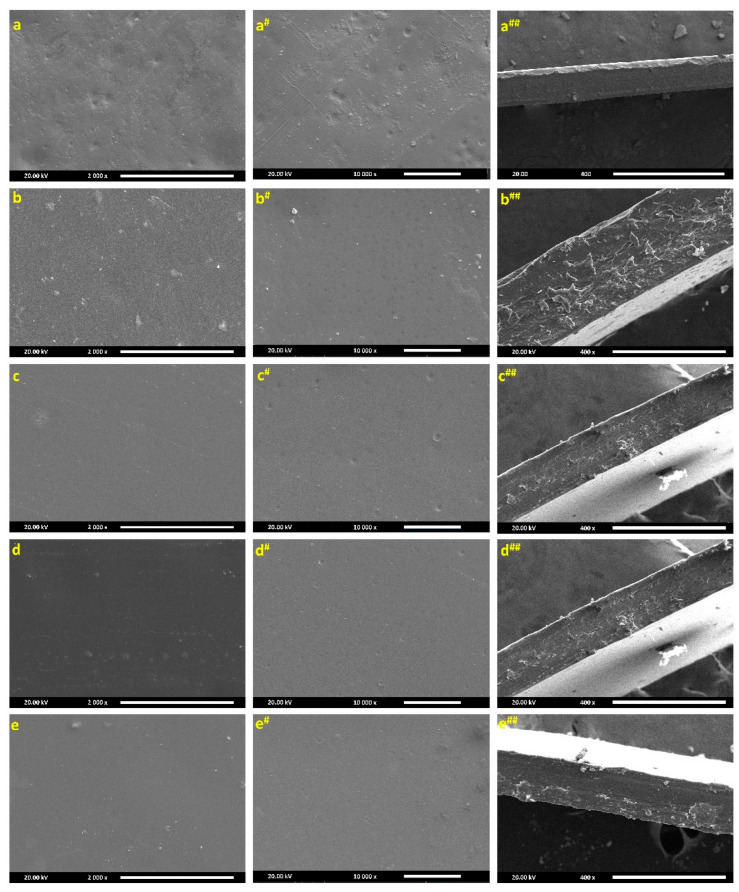
Morphological and Cross-Sectional SEM Images of Pristine PVC and PVC Films Doped with 0.7, 1.0, 2.0, and 3.0 wt% Cr_1.4_Ca_0.6_O_4_.

Figure 5 presents SEM images of pristine and Cr_1.4_Ca_0.6_O_4_-modified PVC films along with their corresponding cross-sectional views. Figure 5a, a^#^, and a^##^ show the pristine PVC film, which exhibits a smooth and homogeneous surface with no visible filler particles, consistent with the absence of any dopants. Figure 5b, b^#^, and b^##^, representing the PVC film doped with 0.7 wt% Cr_1.4_Ca_0.6_O_4_, show a relatively uniform morphology and minimal surface agglomeration, with the cross section revealing a dense and compact structure, indicating good dispersion and strong interfacial interaction. In the case of 1.0 wt% doping (Figure 5c, c^#^, and c^##^), the surface remains relatively smooth, though slight surface roughness and a more stratified cross-sectional structure begin to appear. With 2.0 wt% Cr_1.4_Ca_0.6_O_4_ (Figure 5d, d^#^, and d^##^), minor surface agglomerates are visible, and the cross-sectional image reveals a slightly looser internal structure, possibly due to localized filler clustering. At the highest concentration of 3.0 wt% (Figure 5e, e^#^, and e^##^), more prominent agglomerates appear on the surface, and the cross section indicates a less compact morphology with possible porosity, suggesting limited compatibility and dispersion at high dopant loading. These observations demonstrate that while Cr_1.4_Ca_0.6_O_4_ is generally well-dispersed at lower concentrations, higher loading leads to increased agglomeration, likely due to stronger particle–particle interactions and limitations in the dispersion process. Such morphological differences directly affect the interfacial bonding between PVC chains and dopants, which in turn influence the overall performance of the films.

**Figure 6:**
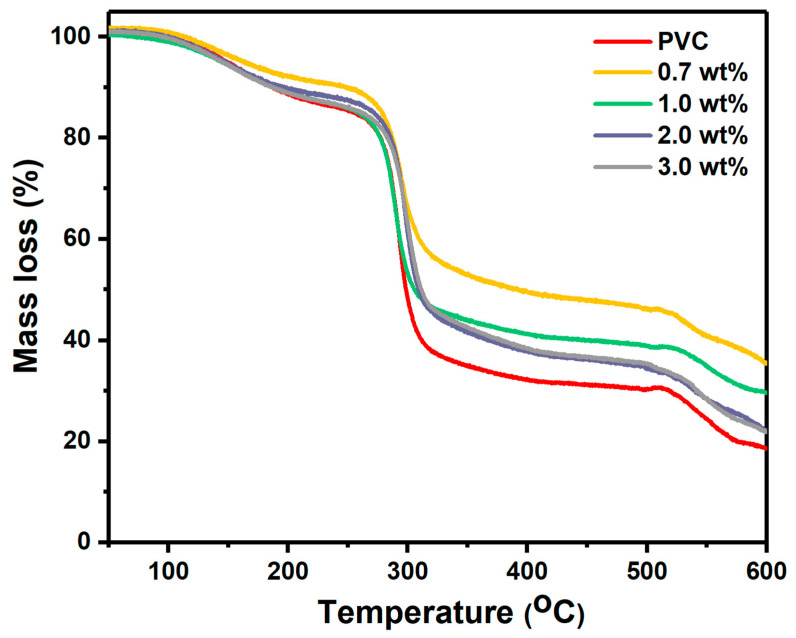
TGA Profiles of Pristine PVC and PVC Films Doped with 0.7, 1.0, 2.0, and 3.0 wt% Cr_1.4_Ca_0.6_O_4_.

Figure 6 shows the TGA measurement in the temperature range of 50–600 °C to investigate the thermal stability of pristine and modified PVC films with Cr_1.4_Ca_0.6_O_4_. Both pristine and modified PVC films with Cr_1.4_Ca_0.6_O_4_ exhibited three degradation stages. The first degradation stage occurred below 180 °C, which was assigned to the solvent evaporation, such as THF [26]. The second degradation stage was observed at 260–330 °C where pristine PVC film has mass loss of 63% and the modified PVC films with 0.7, 1.0, 2.0, and 3.0 wt% Cr_1.4_Ca_0.6_O_4_ have mass loss of 45%, 55%, 56.2%, 55.3%, respectively. The third degradation stage began at 518 °C where pristine PVC film has a mass loss of 17% and modified PVC film with 0.7 wt% Cr_1.4_Ca_0.6_O_4_ has a mass loss of 16.5%. Although the incorporation of Cr_1.4_Ca_0.6_O_4_ shows a moderate improvement in thermal stability, particularly by reducing the mass loss during the main decomposition phase, the enhancement is not highly significant. Among the composites, the 0.7 wt% Cr_1.4_Ca_0.6_O_4_-doped film demonstrates the best thermal behavior, with the lowest total mass loss compared to both pristine and other modified samples. The variation in mass loss percentages among the pristine and modified PVC films at the same temperature values can be attributed to differences in the dispersion, thermal conductivity, and interaction of Cr_1.4_Ca_0.6_O_4_ particles within the PVC matrix. Well-dispersed metal oxides can act as thermal insulators or barriers, delaying degradation by restricting heat and mass transfer, thus reducing decomposition rates [8,27]. To fully understand the stabilization mechanism, further investigations, including FTIR spectral analysis, glass transition temperature (Tg) measurements, and mechanical property testing, are required to evaluate the composite’s potential for engineering applications.

**Figure 7:**
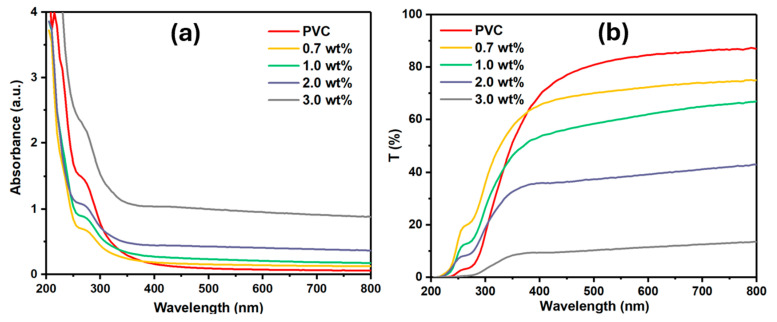
UV–Vis Spectra (**a**) and Transmittance Spectra (**b**) of Pristine PVC and PVC Films Doped with 0.7, 1.0, 2.0, and 3.0 wt% Cr_1.4_Ca_0.6_O_4_.

UV-vis absorption spectroscopy is a straightforward measurement that can be used to identify the optical band gap structure of compounds. The UV-vis absorption measurement of pristine and modified PVC films with varying Cr_1.4_Ca_0.6_O_4_ concentrations (0.7, 1.0, 2.0, and 3.0 wt%) is illustrated in Figure 7a. The Pristine PVC film exhibits an absorption band at approximately 270 nm due to a π→π* transition [17]. The absorption band of PVC has red-shifted when the weight of Cr_1.4_Ca_0.6_O_4_ is increased, due to the interaction between the PVC polymer and the dopants [16]. The interaction induced the charge transfer formation and enhanced the crystallinity of modified polymers. The transmittance, as shown in Figure 7b, decreased gradually as the weight of Cr_1.4_Ca_0.6_O_4_ in the PVC films increased. This is expected since the presence of the dopants reduces the transparency of the PVC film, leading to scattering of incident photons. Although the transmittance decreased when increasing Cr_1.4_Ca_0.6_O_4_, it becomes an advantage for specific applications.

**Figure 8:**
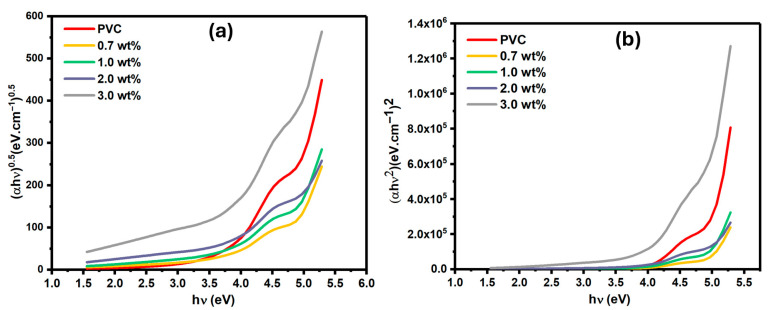
Tauc Plots for Pristine PVC and PVC Films Doped with 0.7, 1.0, 2.0, and 3.0 wt% Cr_1.4_Ca_0.6_O_4_: (**a**) (∝hυ)^0.5^ vs. hν for Indirect Transitions, and (**b**) (∝hυ)^2^ vs. hν for Direct Transitions.

The band gap of synthesized polymer composites relies on the weight and properties of dopants. The Tauc equation can obtain the band gap value within the polymer composites and their electronic transitions (see Equation (2)) [4,28]:

(2) $$\alpha h\upsilon =B{\left(hv-\;E_{g}\right)}^{n}$$

where *α* and *B* represent an absorption coefficient and a constant, respectively, while hυ represents the energy of the incident photon. For direct allowed transitions, *n* = 0.5; however, for indirect allowed transitions, *n* = 2. The values of indirect (E_ind_) and direct (E_dir_) allowed transitions can be evaluated from Figure 8a,b, respectively, when extrapolating a straight line from the edge of the curve to the intercept of the *x*-axis at *hυ* = 0.

**Figure 9:**
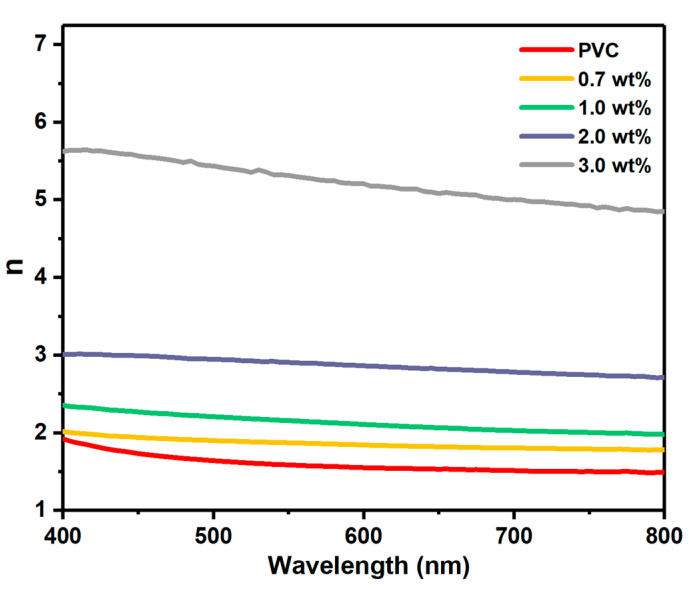
Variation in Refractive Index (n) with Wavelength for Pristine PVC and PVC Films Doped with 0.7, 1.0, 2.0, and 3.0 wt% Cr_1.4_Ca_0.6_O_4_.

The values of E_ind_ and E_dir_ are summarized in Table 1, and it is obvious that the presence of Cr_1.4_Ca_0.6_O_4_ reduced their values. This is evidence of interaction between Cr_1.4_Ca_0.6_O_4_ and PVC, resulting in the creation of localized electronic states that lead to a change in the optical band gap of the polymer due to alterations in its structure [17]. The refractive index (n) of polymer composites is a key parameter for several optoelectronic applications. It relies on several factors, such as the structure of molecules, the properties of dopants, and the thickness of the film [28]. Equation (3) shows the dependency of n on the reflectance, which represents an amount of reflected incident light [4].

(3) $$n=\left(\frac{1+R}{1-R}\right)+\sqrt{\begin{array}{c} \frac{4R}{{\left(1-R\right)}^{2}}-k^{2} \end{array}}$$

where extinction coefficient is *k* = *αλ*/4*π*. The variation in n versus wavelength at different contents of Cr_1.4_Ca_0.6_O_4_ is shown in Figure 9. The n increases directly when increasing the content of Cr_1.4_Ca_0.6_O_4_ because the density of PVC films increases due to condensation of dopants into the large clusters [29]. It is well known that polymer films with a certain range of high refractive index are desirable for optoelectronic applications.

The refractive index can be obtained by the single oscillator model Equation (4), which includes the oscillator energy (E_0_), dispersion energy (E_d_), and transition moments of materials [4].

(4) $${\left(n^{2}-1\right)}^{-1}=\frac{E_{0}}{E_{d}}-\frac{1}{E_{0}E_{d}}\;{\left(h\upsilon \right)}^{2}$$

**Figure 10:**
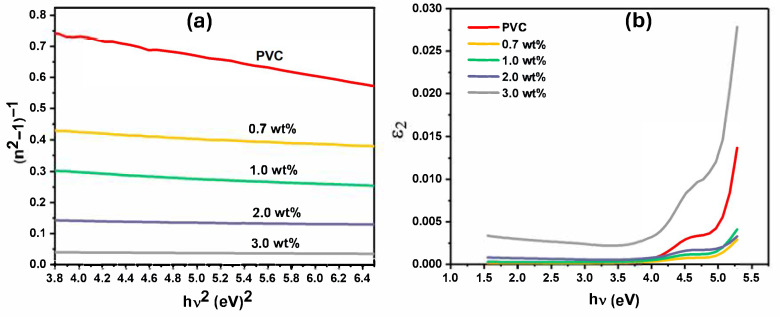
Optical Plots for Pristine PVC and PVC Films Doped with 0.7, 1.0, 2.0, and 3.0 wt% Cr_1.4_Ca_0.6_O_4_: (**a**) (n^2^ − 1)^−1^ vs. *hν^2^* for Single Oscillator Analysis, and (**b**) Imaginary Part of Dielectric Constant (ε_2_) vs. Photon Energy (hν).

The values of E_0_ and E_d_ can be obtained from the plot of (n^2^ − 1)^−1^ versus (hυ)^2^ as shown in Figure 10a. The E_0_ values have increased from 3.98 eV to 5.82 eV when increasing the content of Cr_1.4_Ca_0.6_O_4_ into the PVC film up to 3 wt%; however, Ed values have also increased from 4.08 eV to 107.02 eV as listed in Table 2. The increment in dispersion energy values within the polymer films is attributed to the improved packing of polymer chains during orientation, which also improved intermolecular interactions [30]. The static refractive index of polymer (*n*_0_) at *hυ* = 0 can be determined in the single oscillator model (see Equation (5)) [31]:

(5) $${n_{0}}^{2}=\left(1+\frac{E_{d}}{E_{0}}\right)$$

The addition of Cr_1.4_Ca_0.6_O_4_ to the PVC film drastically increased its *n*_0_ value from 1.42 to 4.75. It is evident that adding Cr_1.4_Ca_0.6_O_4_ as a dopant enhanced the refractive index of the polymer film, qualifying it for a wide range of optical applications. Polymer films with high refractive index are desirable for some of the optoelectronic applications, such as display screens, encapsulation of organic light-emitting diodes (LEDs), micro lenses [32], and modification of plastic lenses [33].

Figure 10b shows the plots of ε_2_ vs. hυ for the pristine and modified PVC films with Cr_1.4_Ca_0.6_O_4_. The real band gap (E_opt_) is determined when extending the linear portion of the curve from its onset to the *x*-axis of the optical dielectric loss (ε_2_ = 2 nk) [34].

The real band gap (E_opt_) indicates the recommended optical transitions of the polymer’s films. For the modified PVC films with Cr_1.4_Ca_0.6_O_4_, the values of E_opt_, as listed in Table 3, are closer to the values of Edir; hence, most optical transitions within the films are direct transitions. The real band gap of modified PVC films with Cr_1.4_Ca_0.6_O_4_ was raised due to the electron-donor nature and polymer backbone conjugation. The rise in E_opt_ is attributed to the enhanced electron-donor nature, polymer backbone conjugation of modified PVC with Cr_1.4_Ca_0.6_O_4_ [35]. The oscillator strength (*f*) of a polymer film depends on its chemical structure, the molecular weight, the thickness of the film, and the aggregation of the polymer molecules. The oscillator strength of the polymer film indicates its suitability for organic solar cells, LEDs, and lasers. The oscillator strength can be obtained by multiplication of oscillator energy (E_0_) and dispersion energy (E_d_) as written in the following equation [36];

(6) $$f=E_{d}E_{0}$$

The values of oscillator strength of pristine and modified PVC with Cr_1.4_Ca_0.6_O_4_ are reported in Table 3. These values increased upon increasing the content of Cr_1.4_Ca_0.6_O_4_ within the PVC films. The electronic structures of molecules within the polymer films are connected to their fundamental chemical structure, therefore directly impacting both their linear and nonlinear optical susceptibilities. The linear susceptibility (χ(1)) illustrates the fundamental interactions of a material with light, such as light refraction and reflection, while the nonlinear susceptibility controls its complex interactions, i.e., harmonic generation and second-order processes. Determining the optical susceptibilities of polymer films is essential for some optical devices. For instance, polymer films with high linear susceptibility can be efficient for optical data storage applications, enabling strong signal modulation. Moreover, polymer films with high nonlinear susceptibility can be beneficial for optical switches and frequency converter applications. Equation (7) is used to calculate the values of linear optical susceptibility χ(1) and third-order nonlinear optical susceptibility χ(3) for pristine and modified PVC with Cr_1.4_Ca_0.6_O_4_ [35];

(7) $$\chi^{\left(1\right)}=\frac{E_{d}/E_{0}}{4\pi },\;x^{\left(3\right)}=6.82\times 10^{-15}{\left(E_{d}/E_{0}\right)}^{4}$$

The calculated values of (χ(1)) and (χ(3)) for pristine PVC and PVC and Cr_1.4_Ca_0.6_O_4_ are summarized in Table 3. The dopants influence the local electric field at the interface between the polymer and the dopants, thereby impacting the third-order nonlinear optical susceptibility. In addition, as a result of the interaction of dopants with polymer molecules, new energy levels are being generated, and thus, the third-order nonlinear optical susceptibility is increased. The chemical structure of polymer films forms the polarizability of its molecules, therefore, impacting the film’s nonlinear refractive index (n_2_) [37]. The n_2_ of polymer films is a valuable parameter in some optical devices. Equation (8) can be utilized to calculate the nonlinear refractive index (*n*_2_) [38];

(8) $$n_{2}=\frac{12\pi x^{\left(3\right)}}{n_{0}}$$

The calculated n_2_ values are listed in Table 3. The inclusion of Cr_1.4_Ca_0.6_O_4_ in the PVC films increased their molecular polarizability, which in turn increased the nonlinear refractive index. This increment was due to the dopant particles—the polymer matrix interfacial interactions resulting in electron distortion surrounding the polymer molecules. Preparing polymer films with an enhanced nonlinear refractive index presents an opportunity to create polymer films with improved optical properties, thereby meeting the requirements for various applications.

## 4. Conclusions

Pristine and modified PVC polymer films with Cr_1.4_Ca_0.6_O_4_ were successfully prepared by the solution casting method. The structure of the polymer films was investigated by using XRD, which confirmed the amorphous nature of PVC films. Moreover, the XRD pattern indicated a tetragonal zircon-type structure of Cr_1.4_Ca_0.6_O_4_ with the average crystallite size of 30.16 nm. FTIR analysis revealed the chemical composition and functional groups present in the PVC polymer films. The XPS survey spectra of pristine Cr_1.4_Ca_0.6_O_4_ and its composites with PVC reveal essential insights into the materials’ surface composition and chemical states. The spectra clearly show peaks corresponding to O1s, Ca2p, and Cr2p, with the Cr2p signals being notably weaker than the other peaks. SEM images showed the surface morphology of pristine and modified PVC polymer films with Cr_1.4_Ca_0.6_O_4_. Despite the appearance of a few minor agglomerations on the surface of PVC polymer films, most parts of the PVC films are homogeneous, and Cr_1.4_Ca_0.6_O_4_ is distributed uniformly within the films. According to the TGA analysis, the incorporation of Cr_1.4_Ca_0.6_O_4_ led to a moderate improvement in the thermal stability of PVC films, with the most notable effect observed at 0.7 wt%. Upon the addition of Cr_1.4_Ca_0.6_O_4_, the linear and nonlinear refractive index and optical susceptibility of PVC films increased accordingly. However, the E_ind_ and E_dir_ decreased when increasing the Cr_1.4_Ca_0.6_O_4_ content within the PVC films. It should be noted that electrical conductivity, an essential property for optoelectronic applications, was not evaluated in this study. Future investigations will focus on characterizing the electrical behavior of these films.

These outcomes make the modified PVC polymer films with Cr_1.4_Ca_0.6_O_4_ convenient for the optoelectronic applications.

## Figures and Tables

**Table 1:** FWHM, d-Spacing, and Crystallite Size of Cr_1.4_Ca_0.6_O_4_ and Cr_1.4_Ca_0.6_O_4_-Doped PVC Films at Various Concentrations Based on XRD Analysis.

Cr_1.4_Ca_0.6_O_3_ (wt%)	FWHM	d-Spacing/nm	Crystallite Size/nm
0.7	0.195	0.271	46.27
1.0	0.960	0.271	16.70
2.0	0.329	0.271	28.90
3.0	0.262	0.272	35.07
100	0.276	0.272	30.52

**Table 2:** Optical Parameters of Pristine PVC and PVC Films Doped with 0.7, 1.0, 2.0, and 3.0 wt% Cr_1.4_Ca_0.6_O_4_.

Cr_1.4_Ca_0.6_O_3_ (wt%)	E_ind_ (eV)	E_dir_ (eV)	E_0_ (eV)	E_d_ (eV)	*n*_0_
0.0	4.67	4.96	3.98	4.08	1.42
0.7	4.64	4.94	5.21	10.55	1.74
1.0	4.59	4.91	4.58	12.62	1.94
2.0	4.43	4.86	5.82	36.82	2.71
3.0	4.36	4.76	4.97	107.02	4.75

**Table 3:** Oscillator Strength, Optical Susceptibilities, Nonlinear Refractive Index, and Optical Band Gaps of Pristine PVC and PVC Films Doped with Cr_1.4_Ca_0.6_O_4_.

Cr_1.4_Ca_0.6_O_3_ (wt%)	*f* (eV^2^)	*χ* ^(1)^ (esu)	*χ* ^(3)^ × 10^−12^ (esu)	n_2_ × 10^−12^ (esu)	E_opt_ (eV)
0.0	16.23	0.081	0.0075	0.199	5.01
0.7	54.96	0.161	0.115	2.49	4.89
1.0	57.8	0.219	0.393	7.64	4.88
2.0	214.29	0.503	10.92	151.91	4.85
3.0	531.89	1.713	1,466.28	11,637.36	4.84

## Data Availability

The original contributions presented in this study are included in the article. Further inquiries can be directed to the author.
